# Ocular Signs Correlate Well with Disease Severity and Genotype in Fabry Disease

**DOI:** 10.1371/journal.pone.0120814

**Published:** 2015-03-17

**Authors:** Susanne Pitz, Gisela Kalkum, Laila Arash, Nesrin Karabul, Andrea Sodi, Sylvain Larroque, Michael Beck, Andreas Gal

**Affiliations:** 1 Department of Ophthalmology, University Medical Centre, Johannes Gutenberg University, Mainz, Germany; 2 Children's Hospital Dr.-Horst-Schmidt-Kliniken, Wiesbaden, Germany; 3 Center for Pediatric and Adolescent Medicine, University Medical Centre, Johannes Gutenberg University, Mainz, Germany; 4 Department of Surgery and Translational Medicine, Eye Clinic, University of Florence, Firenze, Italy; 5 Shire, Zug, Switzerland; 6 Institute of Human Genetics, University Medical Center Hamburg-Eppendorf, Hamburg, Germany; University of Regensburg, GERMANY

## Abstract

Ocular signs in Fabry disease have generally been regarded to be primarily of diagnostic value. We explored whether ocular findings, alone or in particular in combination with the α-galactosidase A gene mutation, have predictive value for disease severity. Data from the Fabry Outcome Survey (FOS), a large, global database sponsored by Shire, were selected for adult patients who had undergone ophthalmological examination. Three ocular signs were assessed: cornea verticillata, tortuous conjunctival and/or retinal vessels, and cataract. Fabry disease severity was measured using FOS Mainz Severity Score Index and modifications thereof. Ophthalmological data were available for 1203 (699 female, 504 male) adult patients with eye findings characteristic of Fabry disease in 55.1%. Cornea verticillata had a similar distribution in women (51.1%) and men (50.8%), whereas tortuous vessels and Fabry cataract were somewhat more frequent in men than in women. Patients with cornea verticillata, selected as the principal ocular sign for this study, had more severe disease (median score, 20.0) versus those without ocular signs (11.0; *P*<0.001). This finding could be confirmed by applying age adjusted severity scores. Moreover, the prevalence of cornea verticillata was significantly higher in patients with null (male, 76.9%; female, 64.5%) and missense (male, 79.2%; female, 67.4%) mutations versus mild missense (male, 17.1%; female, 23.1%) and the p.N215S (male, 15.0%; female, 15.6%) mutations (*P*<0.01). Our analyses show a correlation between the prevalence of ocular changes in Fabry disease and disease severity. Consequently, information on ocular findings and α-galactosidase A gene mutation may help assess the risk for more severe Fabry disease. These observed findings are of notable clinical importance, as Fabry disease is characterized by high clinical course variability and only weak genotype-phenotype correlation at the individual patient level. Further confirmatory studies are needed.

## Introduction

Fabry disease is an X-chromosomal lysosomal storage disorder resulting from deficient activity of the enzyme α-galactosidase A. The progressive deposition of sphingolipids within lysosomes affects virtually every tissue. Progressive cardiomyopathy, nephropathy, and cerebrovascular events are the most important organ complications, contributing to significant morbidity and mortality. Men with untreated Fabry disease die from these organ complications, on average, in the fifth decade of life and women in the seventh decade, reflecting approximately 20- and 15-years loss of life, respectively, compared with normal life expectancies [1,2]. In addition to symptomatic therapy, enzyme replacement therapy (ERT) has been available since 2001 in Europe and 2003 in the United States, and has proven to offer substantial clinical benefits [3,4].

Ocular involvement in Fabry disease is characterized by corneal and lens opacities as well as vascular abnormalities [5–8]. Cornea verticillata manifests as almost pathognomonic corneal deposits. These whorl-like, linear opacities in the inferior part of the cornea have been shown histologically to be located in the epithelium and adjacent anterior stroma [8]. Cornea verticillata does not affect vision, but is the most common ocular sign in Fabry disease; the reported prevalence varies between 44% and 94% among various subpopulations [5,9,10]. Other, but less specific and/or less common findings are diffuse corneal opacity (“corneal haze”), aneurysms of conjunctival vessels, increased tortuosity of conjunctival and/or retinal vessels, and anterior and/or posterior subcapsular lens opacities. Ocular signs have been reported to occur as early as the first decade of life [5]; cornea verticillata has even been described in a 22-week foetus [11].

The Fabry Outcome Survey (FOS), sponsored by Shire, is an international database of patients with Fabry disease; the majority of enrollees are receiving ERT with agalsidase alfa (Replagal^®^; Shire, Lexington, Massachusetts, USA). Data from FOS provide the opportunity to study large numbers of individuals affected by this rare disorder. A previous analysis of FOS data by Sodi et al [12] suggested the possibility that ocular signs might have predictive value for disease severity. Analysis of a subpopulation of the FOS cohort showed a correlation between increased ocular vessel tortuosity, higher values in the disease-specific FOS severity index (FOS Mainz Severity Score Index [FOS-MSSI]), and greater impairment of renal and cardiac function [12].

Ophthalmic findings in Fabry disease have been reported to be more common in pediatric patients with loss-of-function mutations of the α-galactosidase A gene (*GLA*) versus those with genotypes associated with residual enzyme activity [13].

The current analysis was conducted to explore whether correlations between ocular involvement and disease severity could be confirmed in a larger cohort of patients, and whether this correlation is associated with the nature of the gene mutation.

## Materials and Methods

### Patients and study design

At the time of the current analysis, detailed ophthalmological data in the FOS database were available for 1203 adult patients from 83 centers. *GLA* mutation information was available for 836 of these patients (500 women and 336 men). This study was approved by the institutional review board at each center, all patients in this analysis provided written informed consent to participate. Depersonalized data were collected from the FOS database. The prevalence of eye lesions was analyzed by gender and age. Amongst other data, the presence or absence of the following 3 signs were specifically investigated for the present analysis: cornea verticillata, tortuous conjunctival and/or retinal vessels, and cataract.

**Fig 1:**
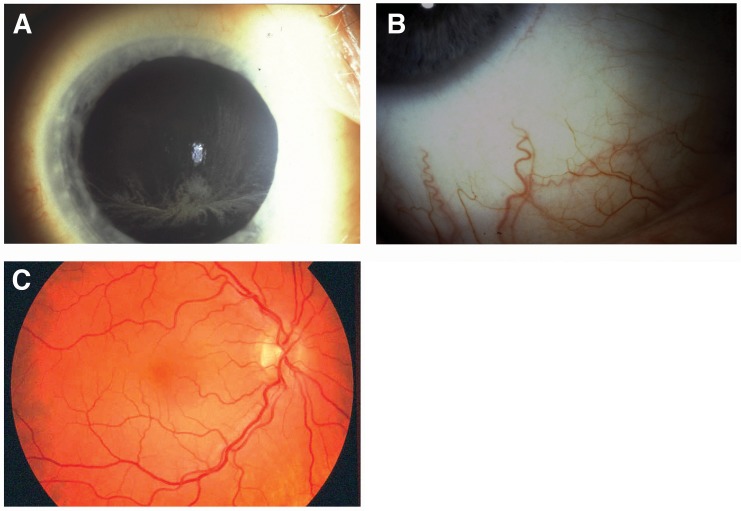
Representative ocular changes seen in patients with Fabry disease. Representative ocular changes include (A) cornea verticillata, (B) increased tortuosity of conjunctival vessels, and (C) increased tortuosity of retinal vessels.

Cornea verticillata (Fig. 1A) and tortuosity or aneurysms of the conjunctival vessels (Fig. 1B) were assessed by slit-lamp examination, and tortuosity of the retinal vessels (Fig. 1C) was assessed by fundoscopy. Fabry cataract was diagnosed during ophthalmic examination (usually with pharmacological mydriasis) by the presence of whitish, spoke-like linear deposits on or near the posterior capsule of the lens, with or without wedge-shaped, radial, cream-colored, granular deposits towards the periphery of the anterior lens capsule. Use of medical mydriasis to facilitate fundoscopic/lens examination was used in the majority of the patients at the discretion of the investigating ophthalmologist.

### Data analyses

Descriptive summary statistics were calculated. For continuous parameters, median and range were recorded; for categorical parameters, numbers and percentages of patients affected were recorded. The Wilcoxon non-parametric statistic was computed for comparing 2 groups in continuous parameters. The Cochran-Mantel-Haenszel statistic with ordinal alternative was computed for detecting trends in proportions of patients split by mutation groups. The parametric linear testing with equal space contrasts was computed for detecting trend in continuous parameters. Statistical analysis was performed using SAS version 9.2 (SAS Institute Inc, Cary, NC). Data collection and analysis were supported by Shire Outcomes Research (Eysins, Switzerland).

The correlation between eye abnormalities and the severity of systemic involvement was investigated. Fabry disease severity was measured using the Mainz Severity Score Index, adapted for use in FOS (FOS-MSSI) [14]. The total FOS-MSSI score has 4 domains: general, neurologic, cardiovascular, and renal, each weighted according to its contribution to the morbidity of the disease. This tool is helpful in scoring each individual’s disease burden in this clinically highly variable disorder [14,15]. Moreover, it allows comparison of disease severity and has been used to study efficacy of treatment [16–18]. The general and renal components have a maximum score of 18 and the neurologic and cardiovascular components have a maximum score of 20 (S1 Table); these individual components are summed into the total MSSI score, which is then classified into mild (<20), moderate (20–40), and severe (>40) clinical categories [14]. The cornea verticillata subscore was removed before calculating the FOS-MSSI and other derived scores.

Fabry disease is a progressive disorder, and the FOS-MSSI score depends on age as well as gender. Therefore, the age-adjusted modified FOS-MSSI (aaFOS-MSSI) has been recommended for the use with the data in FOS [19] to allow comparison of disease severity among subgroups without age or gender as confounding factors; however, this score does not allow comparison between genders. In order to compare data between males and females, we developed a second model of adjustment to calculate an age-related individual FOS-MSSI (ariFOS-MSSI) value [20]. Briefly, a normalised ariFOS-MSSI value (taking into account the age of the patient) was defined by a regression line calculated to obtain the mean FOS-MSSI score for each age group. The difference between a patient’s actual and expected scores provides an ariFOS-MSSI score, with positive scores representing a more severe phenotype than expected and negative scores reflecting a milder one. The ariFOS-MSSI was developed using data from 486 male patients aged >6 to 60 years from before or <3 months after initiating ERT and for whom mutation type and FOS-MSSI were available at baseline (and excluding those with the p.N215S mutation); ariFOS-MSSI scores have previously been shown to significantly correlate with GLA mutation type classified as described below [20]. For the purpose of our analysis, this model was applied to both male and female cohorts, assuming similar correlation between FOS-MSSI score and age in both gender groups.

The information regarding genetic mutations was collected at each of the FOS sites as part of the diagnostic and clinical evaluation of patients and then entered into the FOS database. Genetic mutations were classified into 4 groups by the authors. Null alleles were defined as a *GLA* mutation associated with an absent or non-functional enzyme protein. These included among others out-of-frame rearrangements, in-frame re-arrangements larger than 3 nucleotides, premature termination codons, mutations affecting splice consensus sequences, missense mutations affecting the evolutionarily conserved residues of the active site of the enzyme, and the cysteines involved in proper folding of the protein by establishment of disulfide bridges. Missense mutations were those resulting in polypeptides with an amino acid replacement probably not leading to a null allele. “Mild” missense mutations and the glycosylation mutation p.N215S defined a mutant enzyme with measurable residual activity and were reported in the literature to be associated with an attenuated phenotype.

## Results

Clinical characteristics are shown in Table 1 and S2 Table. As of October 2013, ophthalmological data were available in the FOS for 1203 (699 female and 504 male) adult (≥18 years of age) patients. Of these 1203 patients, 750 were from Europe, 219 were from North America, 81 were from South America, 68 were from Asia, and 85 were from Australia. The median age at diagnosis of Fabry disease was 35 years; the median delay between first symptoms and the diagnosis was 10.5 years. Further, 771 (64.1%) patients were receiving ERT with agalsidase alfa (0.2 mg/kg every other week; median treatment duration, 3.3 years).

**Table 1:** Ophthalmological characteristics and treatment status.

Variable	Patients, n (%)		
	Female	Male	Overall
**Adult FOS patients (age ≥18 y)**			
Adults with ophthalmic examination	699 (58.1)	504 (41.9)	1203 (100.0)
Any eye finding	385 (55.1)	278 (55.2)	663 (55.1)
Cornea verticillata	357 (51.1)	256 (50.8)	613 (51.0)
Tortuous vessels	112 (16.0)	124 (24.6)	236 (19.6)
Fabry cataract	40 (5.7)	40 (7.9)	80 (6.7)
Adults with ophthalmic examination and mutation information available	500 (59.8)	336 (40.2)	836 (100.0)
Any eye finding	297 (59.4)	231 (68.8)	528 (63.2)
Cornea verticillata	280 (56.0)	216 (64.3)	496 (59.3)
Tortuous vessels	86 (17.2)	106 (31.5)	192 (23.0)
Fabry cataract	33 (6.6)	32 (9.5)	65 (7.8)
**Adult FOS patients with ophthalmologic examination and treated at any time with agalsidase alfa**			
No	337 (48.2)	95 (18.8)	432 (35.9)
Yes	362 (51.8)	409 (81.2)	771 (64.1)

FOS = Fabry Outcome Survey.

Treated and untreated patients were analyzed together, as differences in FOS-MSSI scores of patients with and without eye findings were similar irrespective of treatment status (S3 Table), suggesting that treatment had no effect on expression of ocular signs. Statistical comparisons of treated and untreated patient populations were deemed inappropriate because of the substantial mismatch between the 2 cohorts in terms of demographics, event reporting, and disease severity. Ophthalmological data with 5 years of follow-up were available for 181 treated patients; data consisted of a pre-treatment examination and at least 1 yearly investigation. Overall, the eye findings in these patients remained stable during follow-up (S4 Table).

### Prevalence of eye findings

The prevalence of the various eye findings is shown in Table 1. The prevalence of eye findings was higher in the subgroup of patients with available mutation information.

### Correlation of eye findings with Fabry disease severity

**Fig 2:**
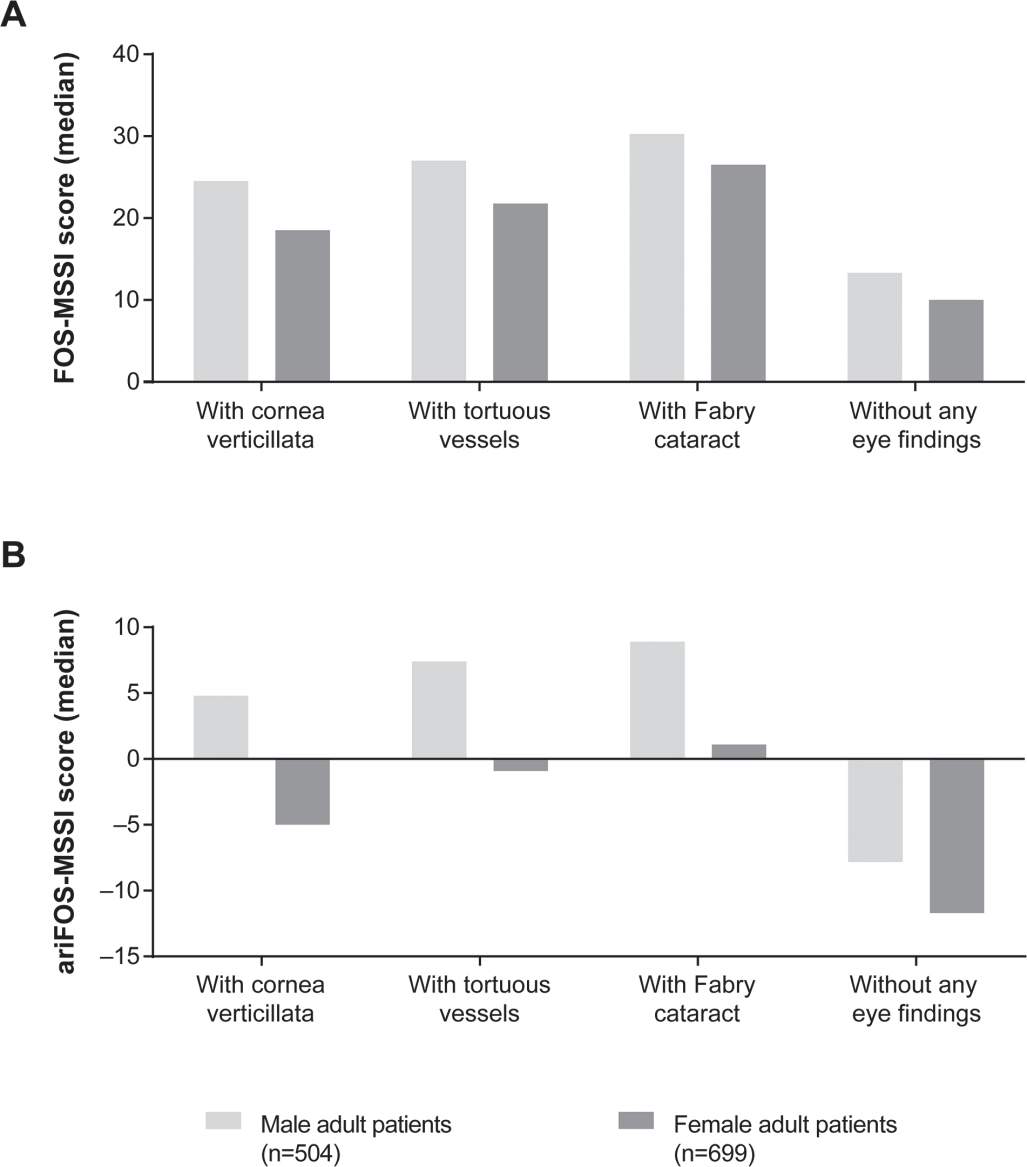
FOS-MSSI and ariFOS-MSSI score, and eye changes. Median (A) FOS-MSSI and (B) ariFOS-MSSI scores in male and female adult patients with and without eye findings. ariFOS-MSSI = age-related individual Fabry Outcome Survey Mainz severity score index; FOS-MSSI = Fabry Outcome Survey Mainz severity score index. The median FOS-MSSI and ariFOS-MSSI scores represent the medians after removing cornea verticillata from the calculation of the FOS-MSSI score.

In the overall patient population, we consistently saw higher FOS-MSSI values in patients with ocular signs of Fabry disease than in patients without these ocular features (S5 Table; Fig. 2A). These scores showed an increase from patients with cornea verticillata to those with tortuous vessels to those with Fabry cataract. It should be noted that FOS-MSSI score increases with age and therefore the age composition of the cohorts may have a considerable effect on the median score values. Therefore, the aaFOS-MSSI was applied and showed a strong positive correlation between the presence of the eye findings under investigation and the FOS-MSSI scores as a measure of overall disease severity (all *P*<0.001; S5 Table). The aaFOS-MSSI is already corrected for gender (as well as age), and thus did not allow for comparison between women and men. Therefore, we applied a second model of age adjustment, the ariFOS-MSSI. The ariFOS-MSSI scores showed a significant correlation of disease severity and the presence of ocular signs, both in women and men (S5 Table, Fig. 2B). The largest median difference in severity scores (12.8 in females and 16.7 in males) was seen between the groups of patients with a Fabry cataract and those without any ocular signs.

**Fig 3:**
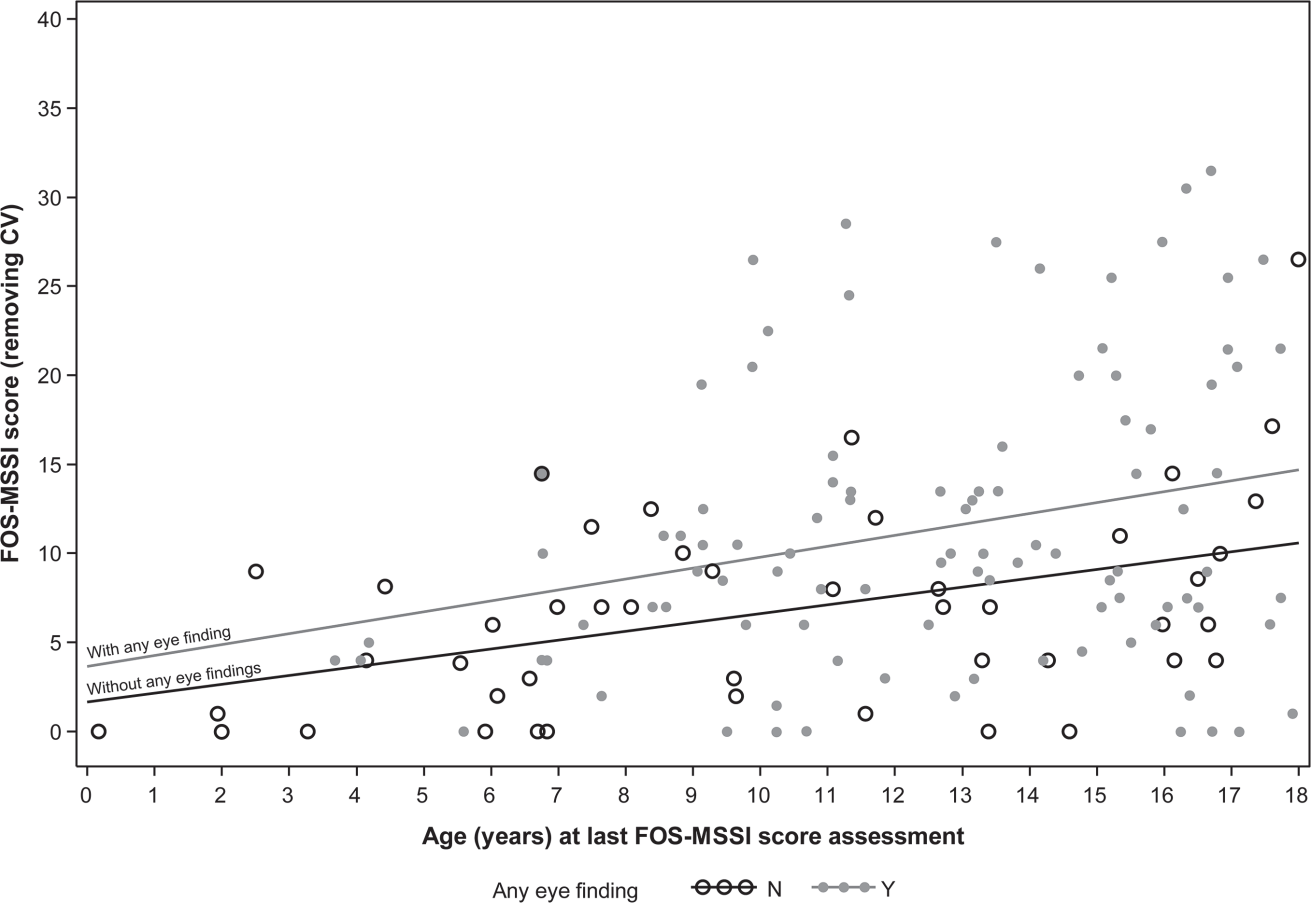
Relationship between disease severity and age in children with and without any eye findings. Scatter plot of FOS-MSSI score versus age (in years) at last FOS-MSSI score assessment, showing regression (trend) lines* for children with and without any eye findings. Children with eye findings (solid grey dots) had more severe disease across all ages than children without any eye findings (open black circles). Disease severity increased with age in both children with and without eye findings. CV = cornea verticillata; FOS-MSSI = Fabry Outcome Survey Mainz severity score index. *Regression lines were determined by the following equations: 1. With any eye findings: FOS-MSSI (removing cornea verticillata) = 3.638976 + 0.613467 × age at FOS-MSSI assessment. 2. Without any eye findings: FOS-MSSI (removing cornea verticillata) = 1.646037 + 0.497238 × age at FOS-MSSI assessment.

The correlation between presence of eye signs and disease severity could also be seen among paediatric patients in the FOS database (n = 145; 65 girls, 80 boys), as shown by the trend lines in a scatter plot (Fig. 3). As seen in adults, disease severity increased with age in children both with and without eye findings, and the differences between the groups remained quite constant over time.

### Correlation between eye changes and the type of mutation

**Fig 4:**
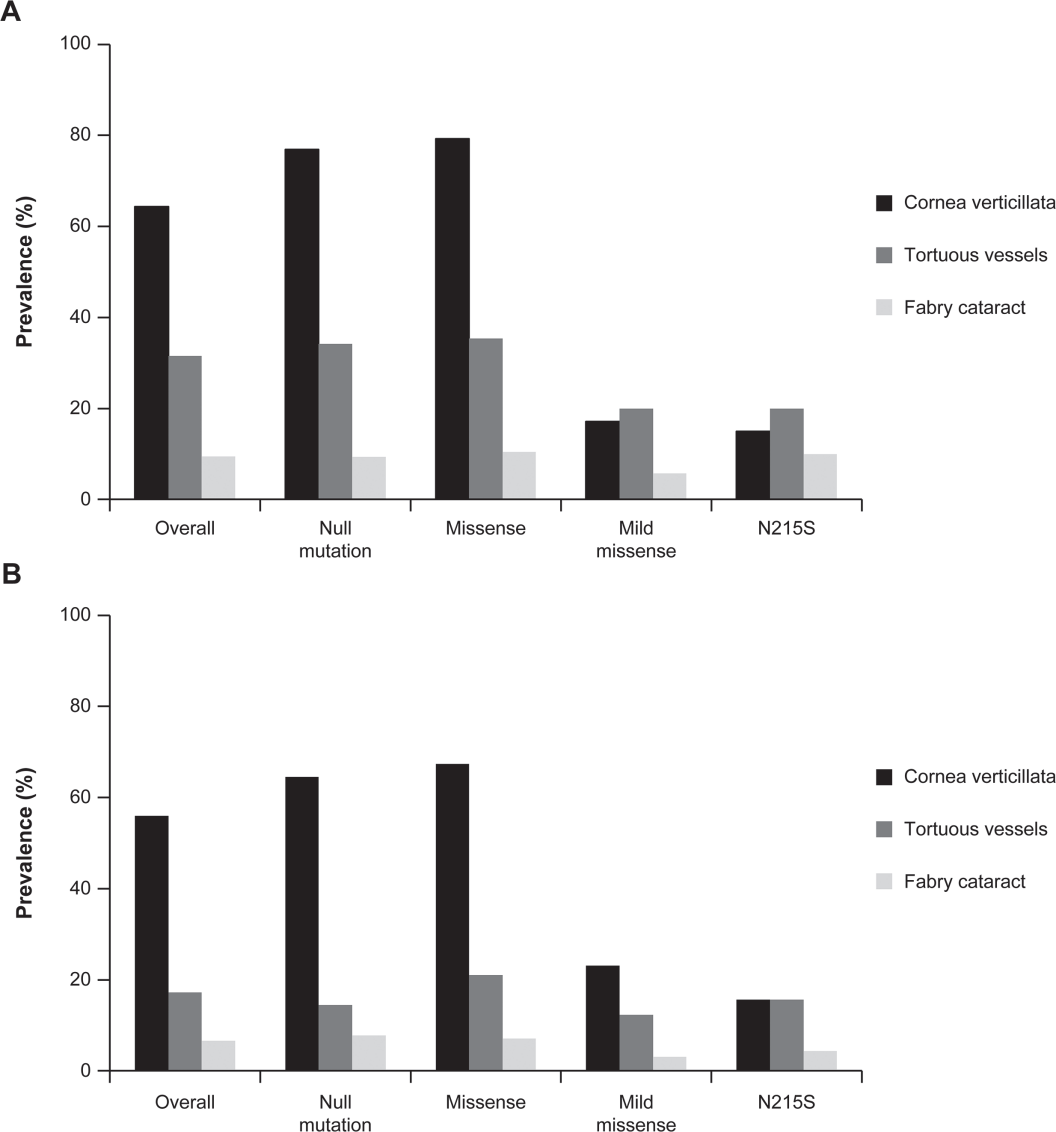
Prevalence of eye findings by type of mutation. Prevalence of eye findings overall (among those patients with mutation information available) and by type of mutation in adult (A) male and (B) female patients.

Patients with null or missense mutations had a higher prevalence of eye findings compared with patients with mild missense or p.N215S mutations. Among the 836 adult patients with ocular examination and mutation information available, cornea verticillata in particular was notably more frequent in men and women with null (76.9% and 64.5%, respectively) or missense mutations (79.2% and 67.4%, respectively) compared with those with mild missense mutations (17.1% and 23.1%, respectively) or the p.N215S mutation (15.0% and 15.6%, respectively; Fig. 4A and B; S6 Table). Similarly, tortuous vessels were more frequent in men with null (34.2%) or missense (35.4%) mutations versus men with mild missense mutations (20.0%) or the p.N215S mutation (20.0%; Fig. 4A; S6 Table).

### Correlation of eye changes, the type of mutation, and disease severity)

**Fig 5:**
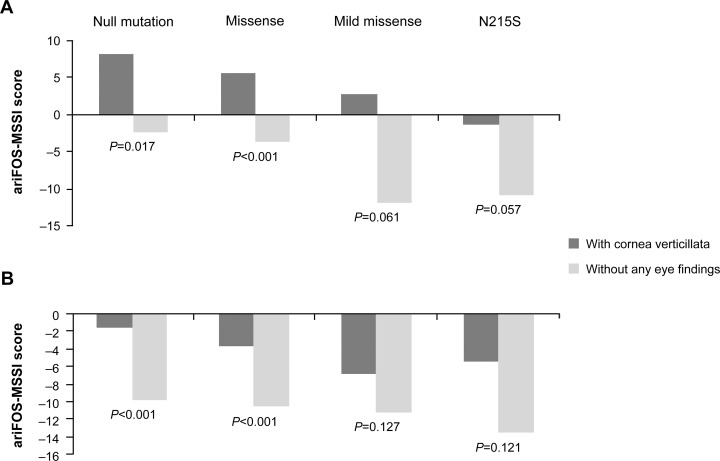
Disease severity score by type of mutation and presence of cornea verticillata. Median ariFOS-MSSI score by type of mutation and presence of cornea verticillata in adult (A) male and (B) female patients. ariFOS-MSSI = age-related individual Fabry Outcome Survey Mainz severity score index.

According to our classification, null mutations and missense mutations result in enzyme protein with no or negligible activity, whereas alpha-galactosidase A produced by a mild missense allele or the p.N215S mutation has a low but still consequential residual activity. Under the assumption that the higher the residual enzyme activity, the less severe the phenotype, we would expect a gradual decrease of the ariFOS-MSSI scores from null mutations towards p.N215S. Such a pattern was indeed found (S7 Table), indicating a positive correlation between the type of mutation and overall disease severity in patients presenting with eye findings versus those with no eye findings. In men and women with null or missense mutations, ariFOS-MSSI scores were significantly higher (*P*<0.001 to *P* = 0.017) among patients with ocular signs (ie, cornea verticillata; S7 Table). Moreover, in male patients with cornea verticillata who had null, missense, or mild missense mutations, ariFOS-MSSI scores were also higher than the corresponding average severity score predicted for age (the zero line in Fig. 5).

## Discussion

Our analysis suggests that the course of Fabry disease among patients exhibiting eye signs is significantly more severe than among those without ocular findings. In consequence, ocular changes (in particular, cornea verticillata) can be of far more value than merely a sign helping to establish the clinical diagnosis. This conclusion is of important clinical relevance, as the assessment of individual patient disease course is significantly hampered by the high clinical variability of Fabry disease.

As the FOS-MSSI in its original description is not corrected for patient age, methods incorporating adjustment for age are mandatory for correct interpretation of pooled data as in a database like the FOS. The use of the gender-independent aaFOS-MSSI permitted disease severity to be compared among various subgroups without confounding by age [19]. We additionally chose to use the ariFOS-MSSI method of age-adjustment [20] to allow comparison between genders. Both scoring systems (aaFOS-MSSI and ariFOS-MSSI) applied in the current study unanimously resulted in significantly higher severity scores for patients with eye changes.

Disease severity and ocular involvement also showed a correlation with different types of mutations: patients from any mutation group presenting with ocular signs had higher disease severity scores compared with patients of the same mutation type but without ocular signs.

According to our data, patients with different mutations showed considerably different prevalences of ocular signs and thus a different “ocular phenotype.” About two-thirds of patients with null and missense mutations showed eye signs compared with only about one-fifth of patients with mild missense or p.N215S mutations. These results confirm previous studies describing a rather high percentage of ophthalmological abnormalities in patients with classical course (usually null alleles or missense mutations) [21,22] and a lower percentage in the so-called late-onset variant of the disease [23]. Of note, this “ocular phenotype” is mostly characterized by the presence of cornea verticillata, the ophthalmological hallmark of Fabry disease. The most frequent eye finding in Fabry disease, cornea verticillata can be easily detected by slit-lamp biomicroscopy and seems to be the sign least influenced by inter- and intra-rater variability [7]. Development of this sign occurs in the early stages of metabolic imbalance, possibly explaining the high prevalence. Cornea verticillata is almost disease-specific (a drug-induced cornea verticillata may be easily ruled out by the patient’s medication history regarding amphiphilic drugs such as amiodarone or chloroquine) with no reported influence of aging or environmental factors, indicating potential usefulness as a biomarker for Fabry disease. Fabry cataract is usually regarded to be pathognomonic; however, the lens opacity described in mannosidosis is strikingly similar [24]. Likewise, increased ocular vessel tortuosity has been reported in healthy individuals as well as in fucosidosis [6], Niemann Pick B disease [25], Gaucher disease [26], and some vascular disorders.

The present investigation supports the conclusion of a previous analysis of FOS data pointing to a correlation of ocular involvement with disease severity. Sodi and co-workers [12] reported a higher FOS-MSSI score, greater progressive deterioration of renal function, and more rapid increase in cardiac size with age in patients featuring tortuous conjunctival and/or retinal vessels. In our opinion, the fact that the present study found a correlation between disease severity and all three ocular signs under investigation is most likely explained by the differing sample sizes. The present analysis was conducted with 1203 adult patients who underwent an ophthalmologic examination to identify specific ocular lesions, whereas the former study by Sodi et al [12] was based on a much smaller sample of 173 patients, with a substantial proportion (33%) <20 years of age. Our data show that this correlation also exists in adult Fabry patients. Another study suggesting a possible relationship between Fabry disease severity and ocular involvement in a pediatric population was conducted by Allen et al [13]. The authors concluded that ophthalmic manifestations in children appear to correlate with loss-of-function mutations. The relationship between higher FOS-MSSI-scores and eye signs is also reported in the pediatric FOS population. Although the FOS-MSSI was not developed to measure disease severity in children with Fabry disease, all data taken together suggest that the “general” and “neurological” parts of the MSSI do apply as well in adults as in children, as these include typical pediatric manifestations of Fabry disease, such as acute and chronic neuropathic pain, hypohidrosis, angiokeratoma, and gastrointestinal symptoms.

Compared with the data reported by Sher et al [6], who found the characteristic whorl-like opacity in 95% of male and 88% of female patients, the 51.0% overall prevalence of cornea verticillata was lower in the current FOS analysis. In contrast, Orssaud and co-workers [10] described less than half of a male patient cohort with this almost pathognomonic corneal opacity. Similar results were obtained for the other ophthalmologic signs of Fabry disease, although the prevalence of Fabry cataract may be slightly underestimated because not all patients underwent the pharmacological mydriasis needed for reliable detection of cataract.

A number of factors may explain these differences. The older literature likely included only cases of Fabry disease with classical clinical and unequivocal laboratory findings, when genetic testing might have been unavailable. Further, females, who have long been regarded as symptom-free “carriers” rather than patients, are and continue to be underreported. In a survey of the literature on the ocular features of Fabry disease, Samiy compiled data from 209 female and 377 male patients, with female patients comprising only about one-third of the total patient population [5]. In FOS, about half of patients are female with ophthalmic data for almost 60%.These differences may explain the variable prevalence of ocular changes in the different studies. Moreover, FOS is an international database and consequently involves a multitude of ophthalmologists. Thus, data collection in such a database likely encompasses a range of different assessment strategies. In our opinion, the large number of patients in the FOS database outweighs this issue. Further, we believe that the composition of the present multicenter patient cohort is less biased than that of the samples investigated in smaller analyses, and therefore we are confident that the prevalences reported here are representative.

Based on the results of this largest group of Fabry patients examined to date with respect to ocular involvement, we conclude that α-galactosidase A mutations combined with eye signs in Fabry disease might be a marker of disease severity. This relationship may facilitate identification of patients who are at risk for more severe disease, as well as earlier initiation of therapy before severe organ damage has occurred. Future prospective studies may further define this relationship and refine the predictive capability of ophthalmic lesions in patients with Fabry disease.

## Supporting Information

S1 Table The Mainz Severity Score Index (MSSI) is a composite of scores for individual signs and symptoms in 4 categories: general, neurological, cardiovascular, and renal [14]. (DOC)

S2 Table Patient demographic and clinical characteristics. (DOC)

S3 Table Mean (SD) ariFOS-MSSI scores for treated and untreated adult male and female patients with ophthalmic examination. (DOC)

S4 Table Timing of first onset of ophthalmological signs during 5 years follow up in 181 treated male and female patients. (DOC)

S5 Table FOS-MSSI, aaFOS-MSSI, and ariFOS-MSSI score, and eye changes. (DOC)

S6 Table Prevalence (n [%]) of eye findings in adult male and female patients by *GLA* mutation type. (DOC)

S7 Table Median (range) ariFOS-MSSI score in adult male and female patients by presence of cornea verticillata and *GLA* mutation type. (DOC)

## References

[1] MacDermot KD, Holmes A, Miners AH. In discussion of: Anderson-Fabry disease: clinical manifestations and impact of disease in a cohort of 60 obligate carrier females. J Med Genet. 2001;38: 769–775. 11732485 10.1136/jmg.38.11.769 PMC1734754

[2] MacDermot KD, Holmes A, Miners AH. Anderson-Fabry disease: clinical manifestations and impact of disease in a cohort of 98 hemizygous males. J Med Genet. 2001;38: 750–760. 11694547 10.1136/jmg.38.11.750 PMC1734761

[3] Breunig F, Weidemann F, Strotmann J, Knoll A, Wanner C. Clinical benefit of enzyme replacement therapy in Fabry disease. Kidney Int. 2006;69: 1216–1221. 16609685 10.1038/sj.ki.5000208

[4] Schiffmann R, Kopp JB, Austin HA 3rd, Sabnis S, Moore DF, Weibel T, et al Enzyme replacement therapy in Fabry disease: a randomized controlled trial. JAMA. 2001;285: 2743–2749. 11386930 10.1001/jama.285.21.2743

[5] Samiy N. Ocular features of Fabry disease: diagnosis of a treatable life-threatening disorder. Surv Ophthalmol. 2008;53: 416–423. 10.1016/j.survophthal.2008.04.005 18572058

[6] Sher NA, Letson RD, Desnick RJ. The ocular manifestations in Fabry's disease. Arch Ophthalmol. 1979;97: 671–676. 106811 10.1001/archopht.1979.01020010327008

[7] Sodi A, Ioannidis A, Pitz S. Ophthalmological manifestations of Fabry disease In: Mehta A, Beck M, Sunder-Plassmann G, editors. Fabry disease: Perspectives from 5 years of FOS. Oxford, UK: Oxford PharmaGenesis; 2006. 21290696

[8] Spaeth GL, Frost P. Fabry's disease. Its ocular manifestations. Arch Ophthalmol. 1965;74: 760–769. 5846554 10.1001/archopht.1965.00970040762005

[9] Nguyen TT, Gin T, Nicholls K, Low M, Galanos J, Crawford A. Ophthalmological manifestations of Fabry disease: a survey of patients at the Royal Melbourne Fabry Disease Treatment Centre. Clin Experiment Ophthalmol. 2005;33: 164–168. 15807825 10.1111/j.1442-9071.2005.00990.x

[10] Orssaud C, Dufier J, Germain D. Ocular manifestations in Fabry disease: a survey of 32 hemizygous male patients. Ophthalmic Genet. 2003;24: 129–139. 12868031 10.1076/opge.24.3.129.15609

[11] Tsutsumi A, Uchida Y, Kanai T, Tsutsumi O, Satoh K, Sakamoto S. Corneal findings in a foetus with Fabry's disease. Acta Ophthalmol (Copenh). 1984;62: 923–931. 6098121 10.1111/j.1755-3768.1984.tb08444.x

[12] Sodi A, Ioannidis AS, Mehta A, Davey C, Beck M, Pitz S. Ocular manifestations of Fabry's disease: data from the Fabry Outcome Survey. Br J Ophthalmol. 2007;91: 210–214. 16973664 10.1136/bjo.2006.100602 PMC1857640

[13] Allen LE, Cosgrave EM, Kersey JP, Ramaswami U. Fabry disease in children: correlation between ocular manifestations, genotype and systemic clinical severity. Br J Ophthalmol. 2010;94: 1602–1605. 10.1136/bjo.2009.176651 20576773

[14] Whybra C, Kampmann C, Krummenauer F, Ries M, Mengel E, Miebach E, et al The Mainz Severity Score Index: a new instrument for quantifying the Anderson-Fabry disease phenotype, and the response of patients to enzyme replacement therapy. Clin Genet. 2004;65: 299–307. 15025723 10.1111/j.1399-0004.2004.00219.x

[15] Lidove O, Joly D, Barbey F, Bekri S, Alexandra JF, Peigne V, et al Clinical results of enzyme replacement therapy in Fabry disease: a comprehensive review of literature. Int J Clin Pract. 2007;61: 293–302. 17263716 10.1111/j.1742-1241.2006.01237.x

[16] Motwani M, Banypersad S, Woolfson P, Waldek S. Enzyme replacement therapy improves cardiac features and severity of Fabry disease. Mol Genet Metab. 2012;107: 197–202. 10.1016/j.ymgme.2012.05.011 22704481

[17] Parini R, Rigoldi M, Santus F, Furlan F, De Lorenzo P, Valsecchi G, et al Enzyme replacement therapy with agalsidase alfa in a cohort of Italian patients with Anderson-Fabry disease: testing the effects with the Mainz Severity Score Index. Clin Genet. 2008;74: 260–266. 10.1111/j.1399-0004.2008.01012.x 18445046

[18] Whybra C, Miebach E, Mengel E, Gal A, Baron K, Beck M, et al A 4-year study of the efficacy and tolerability of enzyme replacement therapy with agalsidase alfa in 36 women with Fabry disease. Genet Med. 2009;11: 441–449. 10.1097/GIM.0b013e3181a23bec 19346951

[19] Hughes DA, Ramaswami U, Barba Romero MA, Deegan P. Age adjusting severity scores for Anderson-Fabry disease. Mol Genet Metab. 2010;101: 219–227. 10.1016/j.ymgme.2010.06.002 20691627

[20] Gal A, Larroque S, Mehta A; on behalf of the FOS investigators. Fabry disease: clinical severity correlates with gene mutation. Presented at: Annual Symposium of the Society for the Study of Inborn Errors of Metabolism; September 4–7, 2012; Birmingham, UK.

[21] Morier AM, Minteer J, Tyszko R, McCann R, Clarke MV, Browning MF. Ocular manifestations of Fabry disease within in a single kindred. Optometry. 2010;81: 437–449. 10.1016/j.optm.2010.02.011 20615758

[22] Morrone A, Cavicchi C, Bardelli T, Antuzzi D, Parini R, Di Rocco M, et al Fabry disease: molecular studies in Italian patients and X inactivation analysis in manifesting carriers. J Med Genet. 2003;40: e103 12920095 10.1136/jmg.40.8.e103 PMC1735554

[23] Lin HY, Huang CH, Yu HC, Chong KW, Hsu JH, Lee PC, et al Enzyme assay and clinical assessment in subjects with a Chinese hotspot late-onset Fabry mutation (IVS4 + 919G—>A). J Inherit Metab Dis. 2010;33: 619–624. 10.1007/s10545-010-9166-7 20821055

[24] Arbisser AI, Murphree AL, Garcia CA, Howell RR. Ocular findings in mannosidosis. Am J Ophthalmol. 1976;82: 465–471. 961797 10.1016/0002-9394(76)90496-7

[25] Rudich DS, Curcio CA, Wasserstein M, Brodie SE. Inner macular hyperreflectivity demonstrated by optical coherence tomography in niemann-pick disease. JAMA Ophthalmol. 2013;131: 1244–1246. 10.1001/jamaophthalmol.2013.2374 24030340 PMC3895486

[26] Shrier EM, Barr CC, Grabowski GA. Vitreous opacities and retinal vascular abnormalities in Gaucher disease. Arch Ophthalmol. 2004;122: 1395–1398. 15364726 10.1001/archopht.122.9.1395

