# Supplementary material for: Ocular Signs Correlate Well with Disease Severity and Genotype in Fabry Disease
Source: PLoS One. 2015 Mar 17;10(3):e0120814. doi: 10.1371/journal.pone.0120814 (PMC4363518; doi:10.1371/journal.pone.0120814)
Supplement: S1 Table — (DOC) [file pone.0120814.s001.doc]

**S1 Table.** The Mainz Severity Score Index (MSSI) is a composite of scores for individual signs and symptoms in 4 categories: general, neurological, cardiovascular, and renal.14

| **Score Category** | **General** | **Neurological** | **Cardiovascular** | **Renal** |
| --- | --- | --- | --- | --- |
| **Signs/symptoms (with subscore range)** | Characteristic facial appearance (0–1)  Angiokeratoma (0–2)  Edema (0–1)  Musculoskeletal (0–1)  Cornea verticillata (0–1)  Diaphoresis (0–2)  Abdominal pain (0–1)  Diarrhea/constipation (0–1)  Hemorrhoids (0–1)  Pulmonary (0–1)  Heart failure (0–4) | Tinnitus (0–2)  Vertigo (0–2)  Acroparesthesia (0–6)  Fever pain crisis (0–2)  Cerebrovascular (0–5)  Depression (0–1)  Fatigue (0–1)  Reduced activity level (0–1) | Changes in cardiac muscle thickness (0–12)  Valve insufficiency (0–1)  ECG abnormalities (0–2)  Pacemaker (0–1)  Hypertension (0–1) | Evidence of renal dysfunction (0–18) |
| **Maximum score** | 18 | 20 | 20 | 18 |
