# Supplementary material for: Ocular Signs Correlate Well with Disease Severity and Genotype in Fabry Disease
Source: PLoS One. 2015 Mar 17;10(3):e0120814. doi: 10.1371/journal.pone.0120814 (PMC4363518; doi:10.1371/journal.pone.0120814)
Supplement: S2 Table — (DOC) [file pone.0120814.s002.doc]

**S2 Table. Patient demographic and clinical characteristics**

| **Variable** | **Patients, n (%)** | | |
| --- | --- | --- | --- |
|  | **Female** | **Male** | **Overall** |
| **Demographic characteristics of adult FOS patients with ophthalmologic examination (n=1203)** | | | |
| Age at FOS diagnosis, median, y | 40.0 | 30.0 | 35.0 |
| Delay between first symptoms and diagnosis, median, y | 9.0 | 11.0 | 10.5 |
| Time from treatment start to last visit, median, y | 3.1 | 3.8 | 3.3 |
| Age at FOS entry, mean (SD), y | 45.2 (15.1) | 41.0 (14.3) | 43.4 (14.9) |
| Gender distribution, n (%) | 699 (58.1) | 504 (41.9) | 1203 (100) |
| White race, n (%) | 638 (92.5)* | 444 (89.5)* | 1082 (91.2)* |
| Country distribution |  |  |  |
| Argentina | 32 (4.6) | 25 (5.0) | 57 (4.7) |
| Austria | 8 (1.1) | 5 (1.0) | 13 (1.1) |
| Australia | 44 (6.3) | 41 (8.1) | 85 (7.1) |
| Belgium | 2 (0.3) | 3 (0.6) | 5 (0.4) |
| Brazil | 12 (1.7) | 12 (2.4) | 24 (2.0) |
| Canada | 134 (19.2) | 78 (15.5) | 212 (17.6) |
| Czech Republic | 3 (0.4) | 1 (0.2) | 4 (0.3) |
| Finland | 3 (0.4) | 0 (0.0) | 3 (0.2) |
| France | 23 (3.3) | 25 (5.0) | 48 (4.0) |
| Germany | 181 (25.9) | 108 (21.4) | 289 (24.0) |
| Hungary | 3 (0.4) | 1 (0.2) | 4 (0.3) |
| Israel | 2 (0.3) | 3 (0.6) | 5 (0.4) |
| Italy | 31 (4.4) | 34 (6.7) | 65 (5.4) |
| Netherlands | 58 (8.3) | 33 (6.5) | 91 (7.6) |
| Portugal | 12 (1.7) | 2 (0.4) | 14 (1.2) |
| Slovenia | 17 (2.4) | 2 (0.4) | 19 (1.6) |
| Spain | 9 (1.3) | 6 (1.2) | 15 (1.2) |
| Sweden | 6 (0.9) | 13 (2.6) | 19 (1.6) |
| Switzerland | 0 (0.0) | 1 (0.2) | 1 (0.1) |
| Taiwan | 28 (4.0) | 35 (6.9) | 63 (5.2) |
| United Kingdom | 91 (13.0) | 69 (13.7) | 160 (13.3) |
| United States | 0 (0.0) | 7 (1.4) | 7 (0.6) |

FOS=Fabry Outcome Survey.

*Percentages based on the patients with data on race available (female, 690; male, 496; overall, 1186).
