# Supplementary material for: Ocular Signs Correlate Well with Disease Severity and Genotype in Fabry Disease
Source: PLoS One. 2015 Mar 17;10(3):e0120814. doi: 10.1371/journal.pone.0120814 (PMC4363518; doi:10.1371/journal.pone.0120814)
Supplement: S3 Table — (DOC) [file pone.0120814.s003.doc]

**S3 Table. Mean (SD) ariFOS‑MSSI scores for treated and untreated adult male and female patients with ophthalmic examination**

|  | **Adult patients with ophthalmic examination** | | |
| --- | --- | --- | --- |
|  | **Treated** | **Untreated** | **Overall** |
| **Male patients*** | **n=409** | **n=95** | **n=504** |
| Patients with any eye findings | 4.6 (10.3), n=196 | 3.3 (9.0),  n=33 | 4.4 (10.1),  n=229 |
| Patients without any eye findings | −6.3 (9.8),  n=84 | −6.7 (11.6),  n=62 | −6.5 (10.6),  n=146 |
| **Female patients*** | **n=362** | **n=337** | **n=699** |
| Patients with any eye findings | −3.3 (10.1),  n=222 | −5.3 (8.8),  n=148 | −4.1 (9.6),  n=370 |
| Patients without any eye findings | −8.3 (9.2),  n=72 | −12.0 (8.3),  n=189 | −11.0 (8.7),  n=261 |

ariFOS-MSSI=age-related individual Fabry Outcome Survey Mainz severity score index.

*Not all patients had ariFOS-MSSI data available.
