# Supplementary material for: Ocular Signs Correlate Well with Disease Severity and Genotype in Fabry Disease
Source: PLoS One. 2015 Mar 17;10(3):e0120814. doi: 10.1371/journal.pone.0120814 (PMC4363518; doi:10.1371/journal.pone.0120814)
Supplement: S4 Table — (DOC) [file pone.0120814.s004.doc]

**S4 Table. Timing of first onset of ophthalmological signs during 5 years follow up in 181 treated male and female patients**

|  | **Patients, n (%)** | | |
| --- | --- | --- | --- |
|  | **Cornea Verticillata**  **n=161** | **Tortuous Vessels**  **n=68** | **Fabry Cataract**  **n=22** |
| Pre-treatment examination | 140 (86.9) | 38 (55.9) | 10 (45.4) |
| Within 1 y of starting treatment | 6 (3.7) | 3 (4.4) | 1 (4.5) |
| Within 2 y of starting treatment | 6 (3.7) | 9 (13.2) | 1 (4.5) |
| Within 3 y of starting treatment | 4 (2.5) | 4 (5.9) | 4 (18.2) |
| Within 4 y of starting treatment | 2 (1.2) | 6 (8.8) | 3 (13.6) |
| Within 5 y of starting treatment | 3 (1.9) | 8 (11.8) | 3 (13.6) |
