# Supplementary material for: Ocular Signs Correlate Well with Disease Severity and Genotype in Fabry Disease
Source: PLoS One. 2015 Mar 17;10(3):e0120814. doi: 10.1371/journal.pone.0120814 (PMC4363518; doi:10.1371/journal.pone.0120814)
Supplement: S5 Table — (DOC) [file pone.0120814.s005.doc]

**S5 Table. FOS‑MSSI, aaFOS-MSSI, and ariFOS‑MSSI score, and eye changes***

|  | **FOS-MSSI score,  median (range)** | | | **aaFOS-MSSI score,  median (range)^†^** | | | **ariFOS-MSSI score,  median (range)** | | |
| --- | --- | --- | --- | --- | --- | --- | --- | --- | --- |
|  | **With eye findings** | **Without any eye finding^‡^** | ***P*-value (Wilcoxon)** | **With eye findings** | **Without any eye finding^‡^** | ***P*-value (Wilcoxon)** | **With eye findings** | **Without any eye finding^‡^** | ***P*-value (Wilcoxon)** |
| **All adult patients (n=1203)** | | | | | | | | | |
| Cornea verticillata | 20.0 (0.0 to 52.5), n=557 | 11.0 (0.0 to 51.5), n=407 | <0.001 | 6.6 (−20.9 to 32.4), n=557 | −1.8 (−28.7 to 30.2), n=407 | <0.001 | −1.5 (−27.4 to 31.0), n=557 | −10.2 (−32.1 to 27.2), n=407 | <0.001 |
| Tortuous vessels | 24.5 (1.5 to 50.5), n=205 | 11.0 (0.0 to 51.5), n=407 | <0.001 | 10.1 (−24.7 to 30.4),  n=205 | −1.8 (−28.7 to 30.2), n=407 | <0.001 | 2.9 (−23.3 to 28.4), n=205 | −10.2 (−32.1 to 27.2), n=407 | <0.001 |
| Fabry cataract | 28.8 (3.0 to 48.5), n=74 | 11.0 (0.0 to 51.5), n=407 | <0.001 | 11.5 (−22.5 to 30.8), n=74 | −1.8 (−28.7 to 30.2), n=407 | <0.001 | 4.4 (−27.4 to 24.9), n=74 | −10.2 (−32.1 to 27.2), n=407 | <0.001 |
| **Male adult patients (n=504)** | | | | | | | | | |
| Cornea verticillata | 24.5 (1.5 to 52.5) n=211 | 13.3 (0.0 to 51.5), n=146 | <0.001 | 6.5 (−20.9 to 32.4), n=211 | −6.4 (−28.7 to 30.2), n=146 | <0.001 | 4.8 (−21.8 to 31.0), n=211 | −7.8 (−28.0 to 27.2), n=146 | <0.001 |
| Tortuous vessels | 27.0 (1.5 to 50.5), n=101 | 13.3 (0.0 to 51.5), n=146 | <0.001 | 9.3 (−24.7 to 30.4), n=101 | −6.4 (−28.7 to 30.2), n=146 | <0.001 | 7.4 (−23.3 to 28.4), n=101 | −7.8 (−28.0 to 27.2), n=146 | <0.001 |
| Fabry cataract | 30.3 (6.0 to 48.5), n=34 | 13.3 (0.0 to 51.5), n=146 | <0.001 | 9.3 (−22.5 to 25.8), n=34 | −6.4 (−28.7 to 30.2), n=146 | <0.001 | 8.9 (−20.3 to 24.9), n=34 | −7.8 (−28.0 to 27.2), n=146 | <0.001 |
| **Female adult patients (n=699)** | | | | | | | | | |
| Cornea verticillata | 18.5 (0.0 to 46.5), n=346 | 10.0 (0.0 to 39.0), n=261 | <0.001 | 6.9 (−16.4 to 30.8), n=346 | −0.2 (−22.6 to 26.5), n=261 | <0.001 | −5.0 (−27.4 to 20.5), n=346 | −11.7 (−32.1 to 15.0), n=261 | <0.001 |
| Tortuous vessels | 21.8 (3.0 to 40.5), n=104 | 10.0 (0.0 to 39.0), n=261 | <0.001 | 10.4 (−10.4 to 28.8), n=104 | −0.2 (−22.6 to 26.5), n=261 | <0.001 | −0.9 (−21.6 to 18.1), n=104 | −11.7 (−32.1 to 15.0), n=261 | <0.001 |
| Fabry cataract | 26.5 (3.0 to 46.5),  n=40 | 10.0 (0.0 to 39.0), n=261 | <0.001 | 13.0 (−16.4 to 30.8), n=40 | −0.2 (−22.6 to 26.5), n=261 | <0.001 | 1.1 (−27.4 to 20.5), n=40 | −11.7 (−32.1 to 15.0), n=261 | <0.001 |

aaFOS-MSSI=age-adjusted modified Fabry Outcome Survey Mainz severity score index; ariFOS‑MSSI=age-related individual Fabry Outcome Survey Mainz severity score index.

*The median FOS‑MSSI and ariFOS‑MSSI scores represent the medians after removing cornea verticillata from the calculation of the FOS‑MSSI score. The n values shown in this table represent the numbers of patients with FOS-MSSI scores available, and thus may differ slightly from the n values shown for ocular sign prevalences in Table 1.

**^†^**aaFOS-MSSI corrects for gender, so male versus female comparisons are not valid.

^‡^Patients without any eye finding (cornea verticillata, tortuous vessels, or Fabry cataract).
