# Supplementary material for: Ocular Signs Correlate Well with Disease Severity and Genotype in Fabry Disease
Source: PLoS One. 2015 Mar 17;10(3):e0120814. doi: 10.1371/journal.pone.0120814 (PMC4363518; doi:10.1371/journal.pone.0120814)
Supplement: S6 Table — (DOC) [file pone.0120814.s006.doc]

**S6 Table. Prevalence (n [%]) of eye findings in adult male and female patients by *GLA* mutation type**

|  | **Overall** | **Null mutation** | **Missense** | **Mild Missense** | **p.N215S** |
| --- | --- | --- | --- | --- | --- |
| **Male adult patients** | | | | | |
| Overall patient number, n | 336 | 117 | 144 | 35 | 40 |
| Cornea verticillata | 216 (64.3) | 90 (76.9) | 114 (79.2) | 6 (17.1) | 6 (15.0) |
| Tortuous vessels | 106 (31.5) | 40 (34.2) | 51 (35.4) | 7 (20.0) | 8 (20.0) |
| Fabry cataract | 32 (9.5) | 11 (9.4) | 15 (10.4) | 2 (5.7) | 4 (10.0) |
| **Female adult patients** | | | | | |
| Overall patient number, n | 500 | 166 | 224 | 65 | 45 |
| Cornea verticillata | 280 (56.0) | 107 (64.5) | 151 (67.4) | 15 (23.1) | 7 (15.6) |
| Tortuous vessels | 86 (17.2) | 24 (14.5) | 47 (21.0) | 8 (12.3) | 7 (15.6) |
| Fabry cataract | 33 (6.6) | 13 (7.8) | 16 (7.1) | 2 (3.1) | 2 (4.4) |
