# Supplementary material for: Ocular Signs Correlate Well with Disease Severity and Genotype in Fabry Disease
Source: PLoS One. 2015 Mar 17;10(3):e0120814. doi: 10.1371/journal.pone.0120814 (PMC4363518; doi:10.1371/journal.pone.0120814)
Supplement: S7 Table — (DOC) [file pone.0120814.s007.doc]

**S7 Table. Median (range) ariFOS‑MSSI score in adult male and female patients by presence of cornea verticillata and *GLA* mutation type**

|  | **ariFOS-MSSI score,*  median (range)** | | |
| --- | --- | --- | --- |
|  | **With cornea verticillata** | **Without any eye finding^‡^** | ***P*-value (Wilcoxon)** |
| **Male adult patients** | | | |
| Null mutation | 8.2 (−12.3 to 31.0)  n=75 | −2.3 (−13.6 to 24.4)  n=10 | 0.017 |
| Missense | 5.7 (−13.2 to 24.9)  n=91 | −3.7 (−28.0 to 14.5)  n=26 | <0.001 |
| Mild missense | 2.8 (−19.7 to 14.7)  n=6 | −11.9 (−20.9 to 14.0)  n=19 | 0.061 |
| p.N215S | −1.3 (−10.6 to 6.3)  n=6 | −10.8 (−20.7 to 3.7)  n=26 | 0.057 |
| **Female adult patients** | | | |
| Null mutation | −1.6 (−25.2 to 20.5)  n=102 | −9.9 (−24.6 to 13.5)  n=48 | <0.001 |
| Missense | −3.6 (−25.8 to 18.1)  n=146 | −10.5 (−26.8 to 15.0)  n=56 | <0.001 |
| Mild missense | −6.8 (−13.5 to 6.1)  n=15 | −11.2 (−31.0 to 10.2)  n=43 | 0.127 |
| p.N215S | −5.4 (−19.1 to 4.0)  n=7 | −13.5 (−26.5 to 6.2)  n=35 | 0.121 |

ariFOS‑MSSI=age-related individual Fabry Outcome Survey Mainz severity score index.

*The median ariFOS‑MSSI scores represent the medians after removing cornea verticillata from the calculation of the FOS‑MSSI score.

**^†^**Patients without any eye finding (cornea verticillata, tortuous vessels, or Fabry cataract).
